# Rapid modulation of interscapular brown adipose tissue mitochondrial activity by ketosis induced by 1,3‐butanediol administration to rats

**DOI:** 10.1096/fj.202401592RR

**Published:** 2024-11-22

**Authors:** Paola Venditti, Gabriella Pinto, Vincenzo Migliaccio, Giuliana Panico, Gianluca Fasciolo, Rita De Matteis, Lillà Lionetti, Pieter de Lange, Stefania Serpico, Gaetana Napolitano, Claudio Agnisola, Angela Amoresano, Assunta Lombardi

**Affiliations:** ^1^ Department of Biology University of Naples Federico II, Complesso Monte Sant'Angelo Naples Italy; ^2^ Department of Chemical Sciences University of Naples Federico II, Complesso Monte Sant'Angelo Naples Italy; ^3^ Department of Chemistry and Biology “A. Zambelli” University of Salerno Fisciano Italy; ^4^ Department of Biomolecular Sciences University of Urbino Carlo Bo Urbino Italy; ^5^ Department of Environmental, Biological, and Pharmaceutical Sciences and Technologies University of Campania “Luigi Vanvitelli” Caserta Italy; ^6^ Department of Science and Technology Parthenope University of Naples Naples Italy; ^7^ Consorzio Interuniversitario Istituto Nazionale Biostrutture e Biosistemi – INBB Rome Italy

**Keywords:** brown adipocyte, ketone bodies, metabolism, mitochondria, thermogenesis

## Abstract

Some studies indicate that brown adipose tissue (BAT) represents a promising target in the fight against dysmetabolic diseases, with indications suggesting it as a potential target for the effects of ketone bodies. We investigate whether the elevation of plasma levels of the ketone body β‐hydroxybutyrate, achieved through the in vivo administration of its precursor 1,3‐butanediol (BD) to rats, could impact interscapular BAT (iBAT) mitochondrial biochemistry and functionality. We examined the effects induced by BD within 3 h and after 2 weeks of treatment. A large‐scale quantitative proteomics approach, coupling liquid chromatography with tandem mass spectrometry (LC–MS/MS) analysis, and Western blot associated with functional studies by respirometry allowed us to evaluate the changes in iBAT mitochondrial protein expression and bioenergetics induced by BD. BD administration increased β‐hydroxybutyrate plasma levels, which correlated with an enhancement in iBAT mitochondrial respiration rate, likely due to the activation of the respiratory chain and uncoupling protein‐1. The proteomic analysis demonstrated that BD influenced the mitochondrial levels of specific subunits belonging to the five respiratory complexes, uncoupling protein‐1, and proteins involved in propanoate metabolism. BD administration also induced lysine β‐hydroxybutyrylation of mitochondrial proteins, including specific subunits of the respiratory chain complexes and uncoupling protein‐1. Most of the BD‐induced effects were observed within 3 h of its administration and persisted/increased after 2 weeks of treatment. In conclusion, by using BD to increase β‐hydroxybutyrate levels, we provide evidence supporting the role of β‐hydroxybutyrate as a signaling molecule capable of rapidly modulating BAT physiology by acting at the mitochondrial level.

## Graphical abstract

Data support the role of β‐hydroxybutyrate (β‐OHB) as a signaling molecule capable of rapidly modulating interscapular brown adipose tissue (BAT) physiology by acting at the mitochondrial level. The in vivo administration of β‐OHB precursor, 1,3‐butanediol (BD), to rats, affects BAT morphology and enhances mitochondrial respiration by activating the respiratory chain and uncoupling protein‐1. The last effect is observed as early as 3 h from BD administration. BD influences mitochondrial proteome patterns and promotes β‐hydroxybutyrylation of lysine residues of mitochondrial proteins.

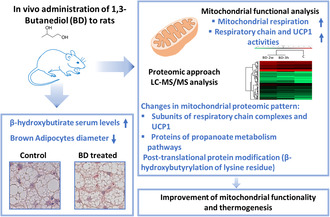

**Abbreviations**
AcAc: acetoacetate
ACN: acetonitrile
ANOVA: one‐way analysis of variance
BAT: Brown adipose tissue
BD: 1,3‐butanediol
BSA: Bovine serum albumin
CID: collision‐induced dissociation
DDA: Data Dependent Acquisition mode
FDR: false discovery rate
G3P: glycerol‐3‐phosphate
GDP: guanosine di‐phosphate
HCOOH: formic acid
HDACI: class I histone deacetylases
HSP‐60: heat shock protein 60
iBAT: interscapular BAT
Kbhb: β‐hydroxybutyrylation
KBs: Ketone bodies
KEGG: Kyoto Encyclopedia of Genes and Genomes
LC–MS/MS: liquid chromatography coupled with tandem mass spectrometry
LFQ: label‐free quantification
mG3PDH: mitochondrial glycerol‐3‐phosphate dehydrogenase
OXPHOS: oxidative phosphorylation complexes
PEP: posterior error probability
PPARs: peroxisome proliferator‐activated receptors
PTM: post‐translational modification
UCP1: Uncoupling protein‐1
VDAC: voltage‐dependent anion channel
β‐OHB: β‐hydroxybutyrate

## 1 INTRODUCTION

Brown adipose tissue (BAT) plays a thermogenic role due to its ability to dissipate the energy obtained by substrate oxidation into heat. BAT expresses high levels of uncoupling protein‐1 (UCP1), a protein localized into the mitochondrial inner membrane that mediates the diffusion of protons from the intermembrane space toward the matrix, a process that is not coupled with the synthesis of ATP.^1^, ^2^ Studies in rodents indicate that to support this energy‐dissipating process, BAT increases the oxidation of glucose, amino acids, and lipids; thus, the activation of BAT thermogenesis can improve metabolic dysregulation, particularly in the context of diet‐induced obesity, characterized by an impaired thermogenic BAT function.^2^ In humans, the presence of ^18^Fluorodeoxyglucose positive BAT appears to be inversely associated with factors like obesity, visceral fat, hyperglycemia, and type‐2 diabetes and seems to be associated with a healthier metabolic phenotype in obesity^3^ and cardiometabolic health,^4^ suggesting that BAT could be a promising target for treating these conditions.^3^, ^4^, ^5^ However, in humans, the limited amount of BAT and the methods used to assess its presence, based on ^18^FDG uptake, cast doubts on its true potential.^6^, ^7^, ^8^ Despite this, significant efforts are being made to identify factors that could influence BAT mitochondrial physiology.

Ketone bodies (KBs) [acetoacetate (AcAc), acetone, and β‐hydroxybutyrate (β‐OHB)] are produced in the liver mitochondria under physiological conditions of enhanced fatty acids oxidation, such as prolonged fasting, calorie restriction, intense physical activity, or even in disease states, such as diabetic ketoacidosis. Hepatocytes do not use KBs as energy substrates, but rather release them into the bloodstream; thus, KBs reach the extrahepatic tissues, where, through ketolysis, they are converted to acetyl‐CoA, which enter the Krebs cycle to produce energy.^9^, ^10^ The metabolic importance of KBs is not restricted to their role as an energy source, since they also play a role as signaling molecules.^11^, ^12^, ^13^, ^14^, ^15^, ^16^ Signaling activated by β‐OHB seems to occur through interaction with membrane receptors coupled to G proteins.^10^, ^11^, ^15^, ^16^ Moreover, KBs can induce post‐translational modification (PTM) of proteins and, through lysine β‐hydroxybutyrylation (Kbhb), β‐OHB is covalently attached to lysine ε‐amino groups. The amount of protein adduct depends on the β‐OHB concentration and involves proteins localized in various cellular compartments, including the nucleus and mitochondria.^14^, ^17^, ^18^, ^19^ In this context, it has been reported that β‐OHB promotes the PTM of histone proteins and the inhibition of class I histone deacetylases (HDACI), thereby modulating gene transcription.^14^, ^17^, ^18^ In addition, several proteins undergo Kbhb modifications in response to experimental conditions that increase β‐OHB in the liver.^19^

KBs have multiple beneficial effects on the whole organism since they counteract inflammation and non‐alcoholic liver disorders, exert cardioprotective effects, and induce a reduction in body weight by increasing energy expenditure and reducing appetite and hunger.^9^, ^10^, ^11^ KBs may improve athletic performance, although conflicting evidence exists regarding this aspect.^9^, ^20^ KBs stimulate mitochondrial biogenesis and enhance antioxidant enzymatic defense systems.^10^, ^14^, ^21^, ^22^ Noteworthy, several physiological conditions characterized by increased KBs serum levels are strictly associated with changes in BAT physiology. In calorically restricted animals, BAT may change its function from an energy‐consuming system to an energy reservoir system,^23^ with reduced thermogenesis.^24^, ^25^ On the other hand, some studies indicate that exercise may activate BAT, with myocyte‐secreted factors (known as “exerkines”) playing a role in such activation.^26^ Ketogenic diet administration to mice for 4 weeks has been reported to enhance UCP1 and mitochondrial proteins.^27^, ^28^ This diet increases plasma‐free fatty acids levels, likely leading to metabolic inefficiency, by the activation of UCP1, mediated by the peroxisome proliferator‐activated receptors (PPARs).^27^, ^28^ Thus, physiological ketosis can induce pleiotropic effects that could mask or confound the roles played by KBs in BAT activation. Studies in which the enhancement in KB_S_ serum levels was achieved by exogenous administration of KBs or their precursor, by pointing to a direct effect of KBs on the tissue, may give different outcomes depending on β‐OHB serum levels reached. Indeed, the administration of Ketone ester D‐β‐hydroxybutyrate‐(*R*)‐1,3 butanediol monoester to high fat diet‐fed obese rats, leading to about a 6‐fold increase in β‐OHB serum level, enhanced tissue UCP1 protein levels and specific markers of BAT activation.^29^ On the other hand, a modest enhancement of β‐OHB (about 1.36 fold), obtained by the administration of β‐OHB in drinking water for 4 weeks to rats, reduced BAT UCP1 levels.^30^ In addition, although studies in the literature suggest that KBs could affect BAT mitochondrial biogenesis,^27^, ^29^ there is still a lack of research on the ability of KBs to influence BAT mitochondrial bioenergetics. Moreover, no studies are available on the possible rapid/short‐term effect elicited by KBs on BAT mitochondria.

Given the role played by both KBs and BAT in regulating energy homeostasis and that of mitochondria in BAT physiology, it is crucial to give further insight into their interaction. To this end, we chose to induce “exogenous ketosis” by in vivo administering 1,3‐butanediol (BD) to rats since the liver rapidly metabolizes it to β‐OHB, and produces dose‐dependent enhancement in β‐OHB levels.^31^, ^32^ The use of BD allows us to shed light on the putative short‐term effect of β‐OHB on BAT mitochondria and, at the same time, to evaluate the mitochondrial adaptation to prolonged exposure to high levels of β‐OHB.

We focused our attention on the ability of BD to affect interscapular BAT (iBAT) mitochondrial biochemistry and functionality. Thus, we conducted a large‐scale quantitative proteomics analysis by coupling liquid chromatography with tandem mass spectrometry (LC–MS/MS) and Western blot analysis to compare the expression of mitochondrial proteins in response to stimulation with BD, and to associate them with changes in mitochondrial functionality, both detected in the isolated mitochondrial enriched fraction. Furthermore, considering that Kbhb is crucial for β‐OHB signaling, we evaluated whether it occurs in BAT mitochondria from BD‐treated rats.

## 2 MATERIALS AND METHODS

### 2.1 Animal treatments

Male Wistar rats weighing 275–300 g (about 10 weeks old) were obtained from Envigo RMS Srl (Udine Italy). Rats were housed in a temperature‐controlled room at 22 ± 1°C under a 12:12‐h light–dark cycle.

In the present study, animals were treated as previously reported.^22^ Three animal groups were used. Group “C” comprised control rats that received a single i.p. administration of a physiological solution at the beginning of the treatment. Group “BD‐2w” comprised rats receiving a single i.p administration of BD at the start of the treatment (2.5 mmol/100 g b.w.), followed by the oral administration of BD dissolved in drinking water (10% w/w) for 2‐weeks. Group “BD‐3h” consisted of rats receiving a single i.p. administration of BD (2.5 mmol/100 g b.w.) and sacrificed 3 h after the injection.

Rats were fed with a standard diet ad libitum (4RF21, Mucedola, Italy) and had free access to water or BD drinking solution that was replaced daily; food, water, and BD drinking solution intake were monitored daily. The amount of BD consumed by rats through drinking was considered to evaluate the animal's total energy intake, and the energy of BD consumed by rats was added to that of food intake. A values of 6 Kcal/gr BD was considered as reported by others.^33^, ^34^, ^35^

BD treatment did not affect fluid intakes (about 2‐0 mL/day for all the groups).

β‐OHB plasma levels were monitored both in control and in BD‐treated rats, after 3 h from either the in vivo administration of BD and vehicle or after 2 weeks of treatment, to assess the efficacy of the treatments. Venus blood samples were obtained from a small cut on the tail, and (D) β‐OHB was detected using a ketone body meter (Glucomen Areo β‐ketone sensor).

At the end of the treatments, rats were anesthetized by i.p. administration of a single dose of sodium Tiopental (40 mg/Kg b.w.) and euthanized by decapitation. Tissues were excised, weighed and iBAT was immediately processed or frozen in liquid nitrogen and stored at −80°C for later analysis.

This study was carried out in agreement with recommendations of the EU Directive 2010/63 for the Care and Use of Laboratory Animals. All efforts were made to minimize the rats' suffering and pain. All animal’ protocols used throughout this study were approved by the Committee on the Ethics of Animal Experiments of the University of Naples Federico II and the Italian Minister of Health (Authorization n° 776/2021‐PR).

### 2.2 Histological analysis

Samples of iBAT were fixed by immersion in 4% formaldehyde in 0.1 M phosphate buffer (overnight at 4°C). The samples were dehydrated in ethanol, cleared, and embedded in paraffin blocks. The tissues were cut into serial 6‐μm‐thick sections; hematoxylin–eosin staining allowed morphological examination. The sections were viewed with a Nikon Eclipse 80i light microscope (Nikon Instruments, Milan, Italy). Images were obtained with a Sony DS‐5M camera connected to an ACT‐2U image analyzer. The average area of adipocytes and the frequency distribution were calculated from three sections per rat and at least four rats for each group (>250 adipocytes counted per group). Adipocyte size distribution is presented as percentage of the total amount of cells. Computed values were imported into Prism 5 (GraphPad Software, San Diego, CA, USA) for data analysis.

### 2.3 Mitochondrial isolation

Immediately after excision, iBAT was deprived of all visible white adipose tissue contaminations and immersed in isolation buffer [220 mM mannitol, 70 mM sucrose, 20 mM Tris–HCl, 1 mM EDTA, 5 mM EGTA, and 0.5% fatty acid‐free bovine serum albumin (BSA), pH 7.4]. Little pieces were homogenized in a Potter‐Elvehjem homogenizer, and the homogenate obtained was centrifuged at 10 000 *g* for 10 min, at 4°C. Floating fat was removed, while the pellet was then re‐suspended in the original volume and centrifuged at 500 *g* for 10 min; the resulting supernatant was centrifuged at 8000 *g* to obtain a mitochondrial pellet. Mitochondrial pellets were washed twice, re‐suspended in a minimal volume of isolation medium, and kept on ice.

The mitochondrial respiration rate was evaluated polarographically using a Clark‐type electrode (Hansatech Instruments Ltd., UK) at 37°C. Isolated mitochondria (0.2 mg/mL) were incubated in a respiration medium made of 80 mM KCl, 50 mM HEPES (pH 7.0), 1 mM EGTA, 5 mM K_2_HPO_4_, and 0.5% fatty acid‐free BSA (w/v). Respiration was started by adding glycerol 3‐phosphate (G3P) (6 mM) or succinate (10 mM). Successively, guanosine diphosphate (GDP) (2 mM) was added to the respiration medium.

### 2.4 Mitochondrial membrane potential

Mitochondria (0.3 mg/mL) were incubated in the respiration medium supplemented with oligomycin (1 μg/mL) and nigericin (80 ng/mL). Respiration rate was determined as described above in mitochondria energized with G3P (6 mM) or succinate (10 mM) as substrate, in the absence and presence of 2 mM GDP. Membrane potential was assayed in 96‐well plates using a fluorescence microplate reader (Tecan Infinity 200‐Pro) using the positively charged dye safranin O, which changes fluorescence intensity linearly proportional to the mitochondrial membrane potential. The safranin O signal for each condition was measured at excitation and emission wavelengths of 540 ± 25 nm and 590 ± 25 nm, respectively, either in the absence of any substrates, in the presence of either G3P (6 mM) or succinate (10 mM) as substrate, or in the presence one of the two substrates and GDP (2 mM). The relative decrease in fluorescent signal upon energization of the mitochondria was proportional to the membrane potential; the results were reported as the absolute magnitude of this change in fluorescence, with larger changes in relative fluorescence units indicating higher membrane potentials.

The respiration rate was plotted as a function of the membrane potential to obtain the kinetic response of the overall reactions involved in the oxidation of substrates and the synthesis of ATP.

### 2.5 Mitochondrial lysate preparation and western blot

Mitochondria were isolated as described above, with isolation buffer supplemented with antiprotease cocktails (Sigma Aldrich). Mitochondria were diluted in RIPA buffer, left on ice for 1 h, and then, centrifuged at 17 000 *g* for 30 min at 4°C. The resulting supernatants were collected, and the protein concentration was determined using Bio‐Rad's DC method (Bio‐Rad Laboratories, RRID:SCR_008426 Hercules, CA).

Primary antibodies used throughout the study were the following: anti‐UCP1 (AB1426; Merk Sigma Aldrich), anti‐mitochondrial glycerol‐3‐phosphate dehydrogenase (mG3PDH) [(GDP2, EPR14259) ab188585 Abcam], a cocktail of antibody used to detect CI‐NDUF88, CII‐SDHB, CIII‐UQCRC2, CIV‐MTCO1, and CV‐ATP VA subunits of oxidative phosphorylation complexes (Total OXPHOS Rodent WB Antibody Cocktail, ab110413, Abcam). Anti‐voltage dependent anion channel (VDAC, ab GTX114187 GeneTex), anti‐heat shock protein 60 (HSP‐60, GTX110089 geneTex), anti‐vinculin (PA5‐8026 Invitrogen, Thermofisher), and anti‐β hydroxyburyl‐lysine (Kbhb, PTM‐1202RM, PTM BIO). The primary antibodies were diluted 1:1000 except for anti‐β hydroxyburyl‐lysine that was diluted 1:500 and anti‐Total OXPHOS that was diluted 1:250.

### 2.6 In solution digestion

For the proteomics analysis, a pool (5 rats) of mitochondrial lysates for each condition (C, BD‐3h, and BD‐2w) was used. For the preparation of the pools, mitochondria were isolated from individual tissues, and some aliquots were frozen in liquid nitrogen. Mitochondrial aliquots from the different tissues used (*n* = 5 for each treatment) were processed simultaneously to obtain individual mitochondrial lysates. After evaluating the protein content of each mitochondrial lysate, the samples (20 μg of protein for each sample) were pooled together. The mitochondrial lysate pool (55 μg of protein) was subjected to a protein extraction protocol with four volumes of cold acetone. The samples were centrifuged for 10 min at 10 000 rpm. The pellets were dried and resuspended in denaturing buffer (6M Urea, 25 mM ammonium bicarbonate). The cysteines were reduced by adding 100 mM DTT (dissolved in denaturing buffer) to the protein suspension and incubated at 60°C for 1 h. After cooling the samples, the reduced cysteines were alkylated with 120 mM iodoacetamide and incubated in the dark for 1 h at room temperature. DTT (100 mM) was added for 1 h at room temperature to quench the alkylation process. For enzymatic digestion, trypsin 1 μg/μL was added to each sample in a ratio enzyme:substrate 1:50. The enzymatic hydrolysis was performed overnight at 37°C in a thermostatic bath. Each sample was treated with the same desalting procedure using stage tips containing 3 layers of 3M Empore C18 membrane. Stage tips were washed with 100 μL 0.1% formic acid (HCOOH) and peptides eluted with 50 μL of 50% acetonitrile (ACN) and, subsequently, with 80% ACN, both acidified with 0.2% HCOOH: The peptide mixture was dried in a vacuum Speed‐Vac, and re‐suspended in 50 μL 5% ACN, 0.2% HCOOH.

### 2.7 LC–MS/MS analysis

The mixture was analyzed by LC–MS/MS on an LTQ Orbitrap XL system (Thermo Fisher) equipped with a nano‐LC Proxeon nanoEasy‐II system. Peptides were fractionated onto a C18 reverse‐phase capillary column (5 μm biosphere, 75 μm ID, 200 mm length) at flow rate of 250 nL/min. A linear gradient from 10% to 60% of eluent B (0.2% formic acid, 95% acetonitrile LC–MS Grade) was used over 200 min. Mass spectrometric analyses were carried out in Data Dependent Acquisition mode (DDA): from each MS scan, spanning from 300 to 1800 m/z, the five most abundant ions were selected and fragmented in the collision‐induced dissociation (CID) modality. Mass tolerance was set at 10 ppm and Orbitrap resolution at 15 000.

### 2.8 Bioinformatics MaxQuant, proteomics data analysis

Raw data files were processed by using MaxQuant software (1.6.8.0 version).^36^ The following parameters were used for raw data processing: trypsin enzyme specificity, 3 missed tryptic cleavages, oxidation of methionine, formation of pyroGlu from N‐terminal glutamine (Q) or glutamic acid (E), β‐hydroxybutyryl‐lysine as variable modifications and cysteine (C) carbamidomethylation as a fixed modification. Identification parameters included minimum peptide length of 6 amino acids, minimum of 1 peptide (both razor and unique peptide). Peptide tolerance of 10 ppm and fragment mass tolerance of ±0.2 Da. All proteins were filtered according to a false discovery rate (FDR) of 0.01% applied both at peptide and protein levels and a maximum peptide posterior error probability (PEP) of 1. The derived peak list generated by Quant.exe (the first part of MaxQuant) was searched using the Andromeda search engine integrated into the MaxQuant against the specific fasta file of *Rattus Norvegicus* obtained from the UNIPROT web site.

### 2.9 Data processing

MaxQuant output files were subsequently processed using Perseus (RRID:SCR_015753) (version 1.6.8.0)^37^ software platforms. An experimental design template was used to get merged replicate experiments (each data set contained two technical replicates) into a single column containing all the proteins into every sample. Contaminants, reverse, and only identified by site hits were filtered out. Expression values of label‐free quantification (LFQ) intensity were log2 transformed and only the protein rows containing a minimum of 2 valid values were maintained within Perseus matrix. Missing values were replaced by random numbers drawn from a normal distribution with a width of 0.3 and a down shift of 1.8. The complete list of proteins (332‐dataset) included UniProt id, gene name, protein name, score, and sequence coverage % (Supplementary dataset, File S2). Finally, the proteins were grouped for BD/C LFQ intensity ratio and ordered for upregulated (>1.2 fold change) and downregulated (<0.83 fold change).

### 2.10 String and Kyoto Encyclopedia of Genes and Genomes (KEGG) pathways software

The STRING software (RRID:SCR_005223) was used to integrate all known and predicted associations between regulated proteins, including both physical interactions as well as functional associations.^38^ A full STRING network (the edges indicate both functional and physical protein associations), a medium confidence of 0.4, and k‐means clustering (network is clustered to a specified number of clusters) were set.

KEGG enrichments were obtained by String and by the KEGG database (RRID:SCR_012773)^39^ by converting Uniprot id into KEGG annotation (genome annotation database).

### 2.11 Statistical analysis

Functional data and Western blots were analyzed with the GraphPad Prism RRID:SCR_002798 software system version 6. Student's *t*‐test or one‐way analysis of variance (ANOVA), followed by the “Tukey correction for Multiple Comparisons” post‐hoc test was used to establish the statistical significance of the differences between experimental groups. Differences were considered statistically significant at *p* < .05.

## 3 RESULTS

### 3.1 Administration of 1,3‐butanediol to rats enhances plasma β‐OHB levels and affects rats' body weight gain, adipose tissue weight, and iBAT morphology

**FIGURE 1:**
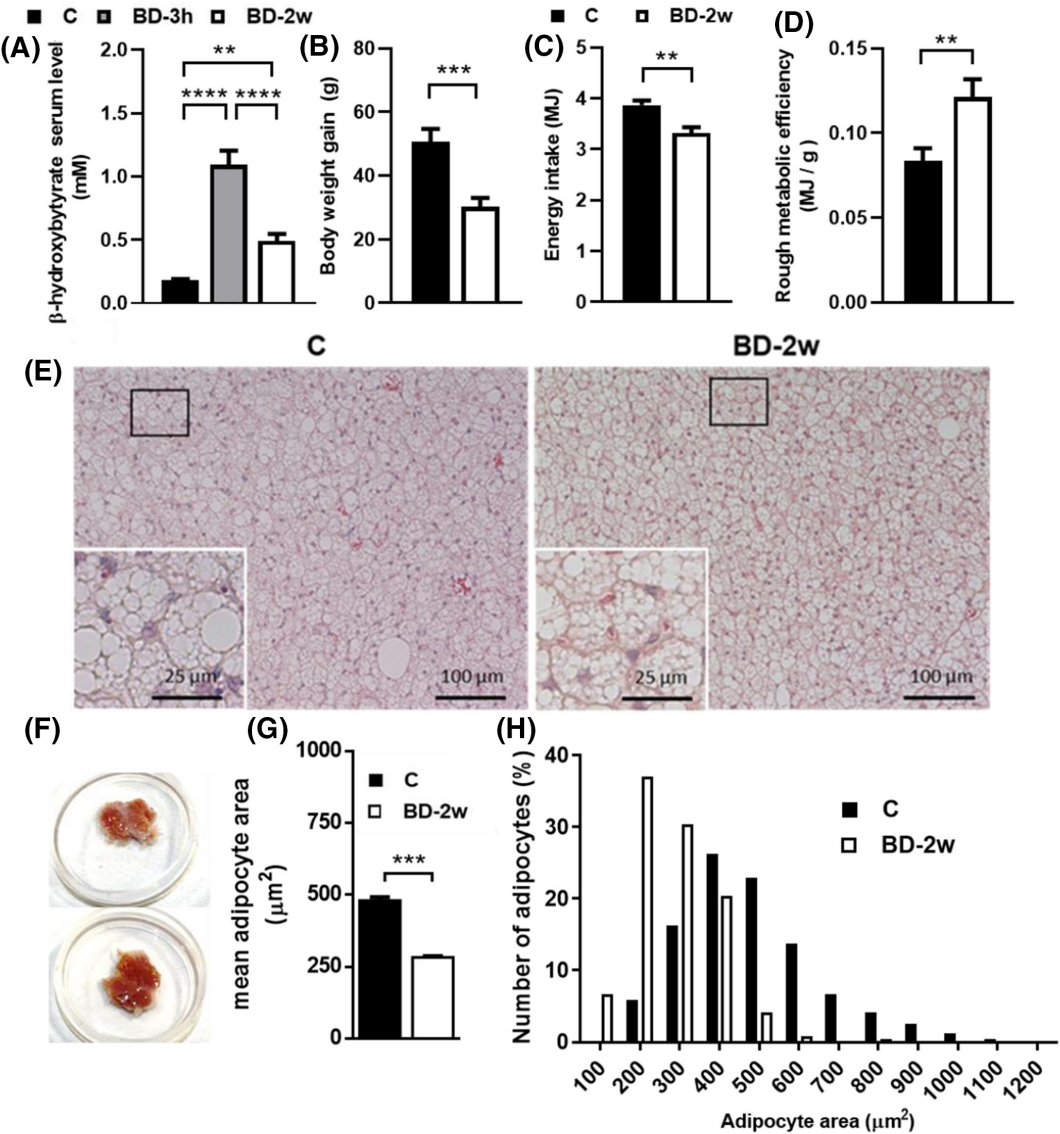
Effect of BD administration on β‐OHB plasma levels, rats' parameters, and iBAT morphology. (A) β‐OHB plasma levels, (B) body weight gain, (C) energy intake, (D) rough metabolic efficiency. Values represent mean ± SEM of 13–14 animals. (E) iBAT hematoxylin & eosin staining; inset represents high magnification of the frame areas, (F) tissues explant, (G) mean cell size, and (H) cell size distribution analysis of iBAT from C and BD‐2w rats. **p < .01; ***p < .001; ****p < .0001.

The in vivo administration of BD was effective in increasing β‐OHB plasma levels. Within 3 h from the i.p. administration (BD‐3h group), β‐OHB serum levels were about 6 fold than C. When the i.p. administration was followed by 2 weeks of BD administration in drinking water (BD‐2w group), β‐OHB levels were ~2.5 fold that of C (Figure 1A). These data confirm the effectiveness of the treatments and our previous data.^22^

Due to the fast increase in β‐OHB plasma levels observed following i.p. BD administration, group BD‐3h allows us to evaluate the rapid response of iBAT mitochondria to a β‐OHB enhancement. Maintaining high levels of β‐OHB for 2 weeks in the BD‐2w group enables us to assess the long‐term adaptation of iBAT mitochondria to high β‐OHB exposure.

At the beginning of the treatment, all the rats showed similar body weights (average of 286 gr). At the end of 2 weeks of treatment, the BD‐2w group gained less weight than the control (−50%) (Figure 1B). During the 2 weeks of treatment, BD‐2w had a lower energy intake (−15%) compared to C (Figure 1C); additionally, the ratio between energy intake and body weight gain (i.e. an index of the rough energy efficiency) was significantly enhanced in BD‐2w versus C (Figure 1D), thus confirming what already reported in our previous paper.^22^ Whole animal VO2, detected on a subset of animals, was enhanced by BD treatment for two weeks (Figure S1). BD influences the contribution of adipose tissues to the total rat weight. The contribution of iBAT was slightly but significantly enhanced by 13%, and that of gonadal white adipose tissue was reduced by about 18% in BD‐2w compared to C. The contributions of liver, skeletal muscle, and heart weight were not affected by BD treatment (Table 1).

**TABLE 1:** Effect of BD treatment for 2 weeks on the contribution of tissues to the rat weight.

	C	BD‐2w
Liver weight/body weight × 100	3.38 ± 0.09	3.15 ± 0.08
Heart weight/body weight × 100	0.27 ± 0.01	0.28 ± 0.01
Skeletal Muscle‐Tibialis weight/body weight × 100	0.25 ± 0.03	0.29 ± 0.04
Skeletal Muscle‐Soleus weight/body weight × 100	0.054 ± 0.002	0.054 ± 0.003
Interscapular Brown Adipose Tissue weight/body weight × 100	0.087 ± 0.006	0.114 ± 0.008*
Gonadal White Adipose Tissue weight/body weight × 100	1.37 ± 0.08	1.13 ± 0.06*

*Note*: Results represent the mean ± SEM of 14–13 different animals.

* *p* < .05 versus C.

Isolated iBAT tissues from BD‐2w rats appear darker in color. Histological analysis of iBAT parenchyma showed typical multilocular cells that in the BD‐2w group presented a reduced average size. Indeed, after 2 weeks of BD treatment, a decrease in the percentage of large adipocytes was observed associated with a concomitant increase in the percentage of small adipocytes (Figure 1E–H).

### 3.2 Proteomics analysis of iBAT mitochondrial lysates

**FIGURE 2:**
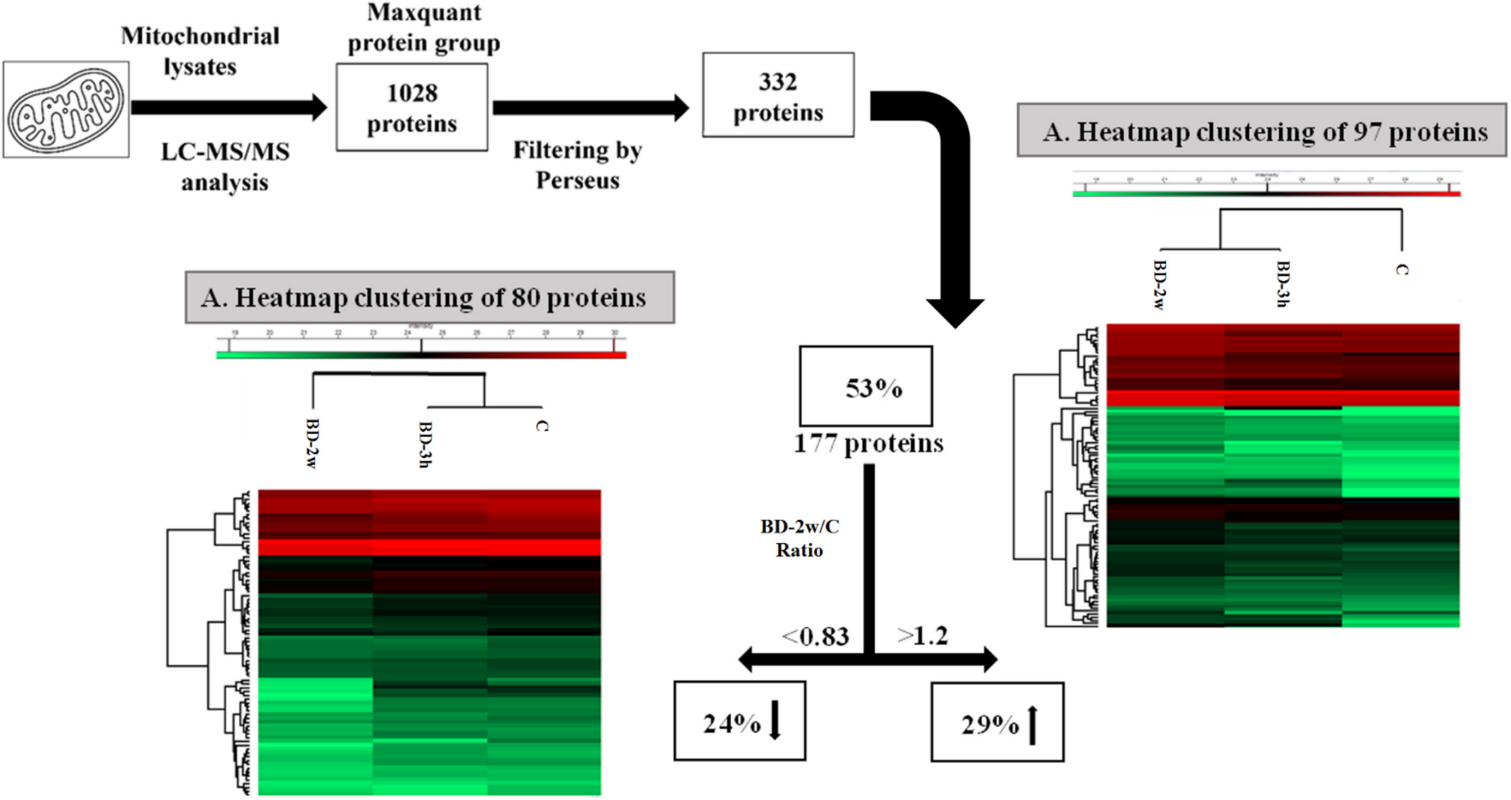
Filtering steps for the selection of a dataset of significant proteins. Heat map hierarchical clustering of 80 down‐regulated proteins (A) and 97 up‐regulated proteins (B) detected along the C, BD‐3h, and BD‐2w. Each value is the result of two technical replicates obtained by a pool of 5 rats.

Proteins obtained by a pool of iBAT mitochondrial lysates were subjected to a classical in‐solution digestion for a large‐scale shotgun bottom‐up approach performed by LC–MS/MS analysis. Data obtained from high‐resolution mass spectrometry requires a powerful platform with quantitative proteomics software to avoid erroneous protein identification and to obtain statistically reliable quantitative information. In our experiment, MS/MS raw data were processed by using MaxQuant, a software useful for protein identification and LFQ toward the comparison of precursor ion peaks across runs, allowing reproducible protein measurements. The protein group file was loaded on Perseus software to visualize and filter proteins according to the “Material and methods” steps. A total of 1028 proteins was identified by using MaxQuant stringent criteria of threshold based on a target decoy database approach and by setting a value of 0.01 FDR at the peptide spectrum match level, corresponding to a 99% confidence score. Approximately 33% of proteins were selected by the steps of Perseus filtering according to the criteria reported in the material and method section, allowing a dataset of 332 proteins (Figure 2).

Within the 332‐dataset, further filtering was performed to chase those proteins with marked dysregulation along the samples by comparing LFQ intensity of BD‐2w versus the C sample. More than 50% of proteins displayed a dysregulation due to the fold change (ratio of LFQ intensity of BD vs. C) higher than 1.2 times or less than 0.83 times. The hierarchical clustering heatmap performed by using Perseus showed a visual representation of the relative expression levels of the 177 proteins, suggesting a strong clustering between C and BD‐3h because of an intermediate behavior of protein expression in BD‐3h. Interestingly, the heatmap of exclusive up‐regulated proteins displayed a change of clustering by reducing the distance between BD‐2w and BD‐3h, thus suggesting that the changes in the abundances of up‐regulated proteins of BD‐2w and BD‐3h resulted to be more similar when compared to C samples (Figure 2, panel A). On the contrary, BD‐2w resulted to be more distinct from the other two samples in the heatmap of down‐regulated proteins highlighting different changes in protein abundance following the 2w stimulation (Figure 2, panel B).

Protein name, UniProt Id, gene name, sequence coverage [%], score, and the ratios of BD‐2w/C and BD‐3h/C LFQ intensity are reported in Supplementary data (File S2).

### 3.3 BD treatment affects the levels of proteins involved in oxidative phosphorylation: Results from proteomic analysis and western blot

**FIGURE 3:**
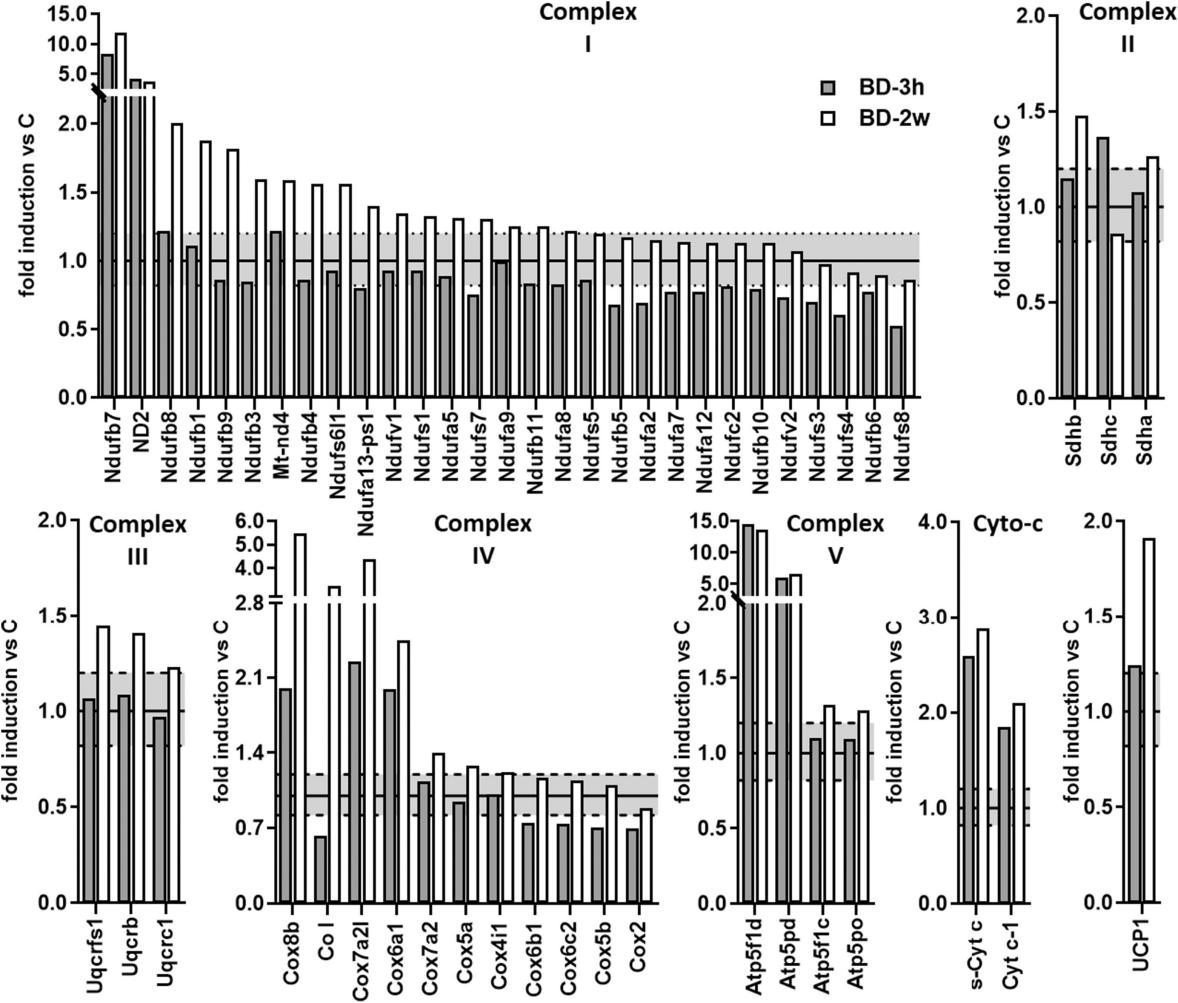
Effect of BD on mitochondrial respiratory chain complexes subunits and UCP1 protein levels. Relative abundance of iBAT mitochondrial proteins determined by the label‐free proteomics approach. Data are summarized for proteins that can be assigned to respiratory chain complexes. Gene names for subunits that have changed have been reported. The values with a fold >1.2 are an expression of upregulation, whereas those with a fold <0.82 are indicative of a downregulation. The upper dotted line represents the value 1.2, and the lower dotted line represents the value 0.82. Each value is a result of two technical replicates obtained by a pool of 5 rats.

Among the up‐regulated proteins, 35% were mitochondrial proteins involved in oxidative phosphorylation. All mitochondrial proteins involved in the oxidative phosphorylation were picked out from the String analysis where functional and physical protein associations were visualized from a big network of lines and edges, obtained from a value of 0.4 interaction score (medium confidence) (Figure S2A). After converting UniProt id codes into the relative KEGG annotations, the results displayed that the proteins belong to the five mitochondrial respiratory complexes as reported in colored boxes highlighted within the KEGG oxidative phosphorylation (Figure S2B). Figure 3 illustrates the change in protein expressions of subunits belonging to the five respiratory complexes, as well as of UCP1 due to BD treatments. A significant fold change greater than 2 was observed for some proteins when analyzing BD‐2w versus C or BD‐3h versus C.

In Complex I, seventeen subunits were upregulated in the BD‐2w group, while none of the detected subunits were downregulated. Notably, ND2, Ndufb7, and Ndufb8 showed the most changes. In the BD‐3h group, four subunits were upregulated (with Ndufb7 and ND2 being the most affected), whereas thirteen subunits were downregulated. In Complex II, peptides from three different subunits were detected by MS. Among them, two subunits (Shdha and Shdb) were upregulated in the BD‐2w group versus C, and one subunit (Shdhc) was upregulated in the BD‐3h group versus C. Other than enhancing complex II subunits, treatment with BD for 2 weeks induced a 3.8‐fold enhancement of Succinate dehydrogenase assembly factor 1 (Sdhaf1), a protein involved in the assembly of the complex, compared to C.

For what Complex III was concerned, upregulation of three subunits was observed in the BD‐2w group versus C, whereas no changes were observed in the BD‐3h group. In Complex IV, seven subunits were upregulated in the BD‐2w group. Notably, CoI, Cox6a1, and Cox8b showed a fold change of BD‐2w/C greater than 2. In the BD‐3h group, upregulation of Cox8b, Cox7a2l, Cox6a1 was also observed, with a fold change BD3‐h/C of about 2. A downregulation of CoI, Cox6b1, Cox6c2, Cox5b, Cox2 subunits was observed in the BD‐3h group compared to C.

In Complex V, four subunits were upregulated in the BD‐2w group versus C, with Atp51fd and Atp5pd showing a fold change greater than 2. Similar stimulation was observed in the BD‐3h group compared to C. In both BD‐2w and BD‐3h groups, increases in Cytochrome c subunits (s‐Cyt c and Cyt‐c1) were detected compared to C.

**FIGURE 4:**
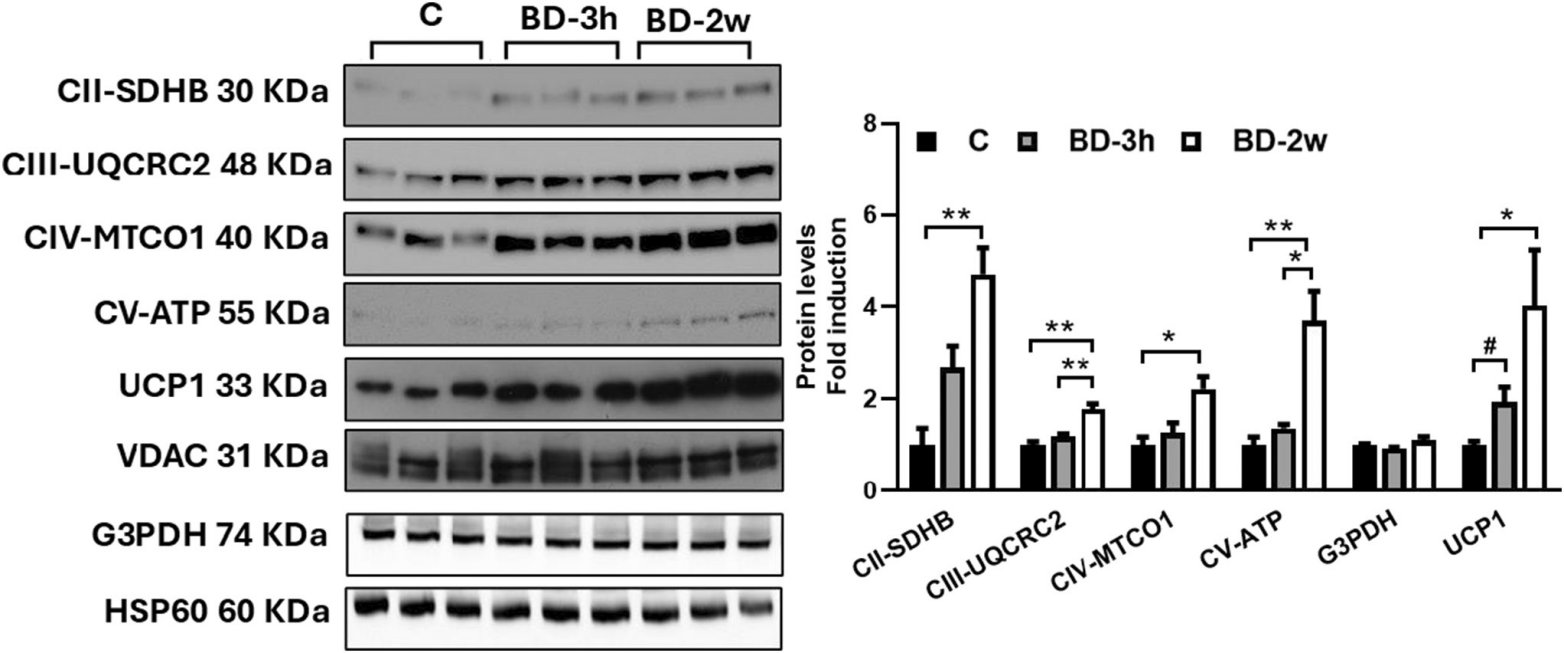
Representative Western blots of respiratory complexes subunits (CII‐SDHB, CIII‐UQCRC2, CIV‐MTCO1, and CV‐ATP VA), mG3PDH, and UCP1 detected in isolated mitochondria from iBAT of C, BD‐2w, and BD‐3h rats (15 μg of protein/mouse/lane). VDAC or HSP60 was used as the loading control. Histograms represent the quantification of data. Data were normalized to the value obtained for C animals, set as 1. Values represent the mean ± SEM of 3 or 6 different rats. *p < .05, **p < .01; ^#^ns by ANOVA, *p* = .018 by *t*‐test.

Moreover, the MS analysis also revealed a progressive increase in UCP1 protein levels in the BD‐3h and BD‐2w groups compared to C (Figure 3). These data were also obtained by Western blot analysis (Figure 4).

Additionally, Western blot analysis showed that BD treatment significantly enhanced the levels of specific subunits of mitochondrial respiratory complexes, whereas it did not affect mG3PDH protein levels (Figure 4). These data are in line with proteomic ones.

### 3.4 BD affects levels of proteins involved in Propionyl CoA‐metabolism and Succinyl‐CoA formation and ketone bodies metabolization

**FIGURE 5:**
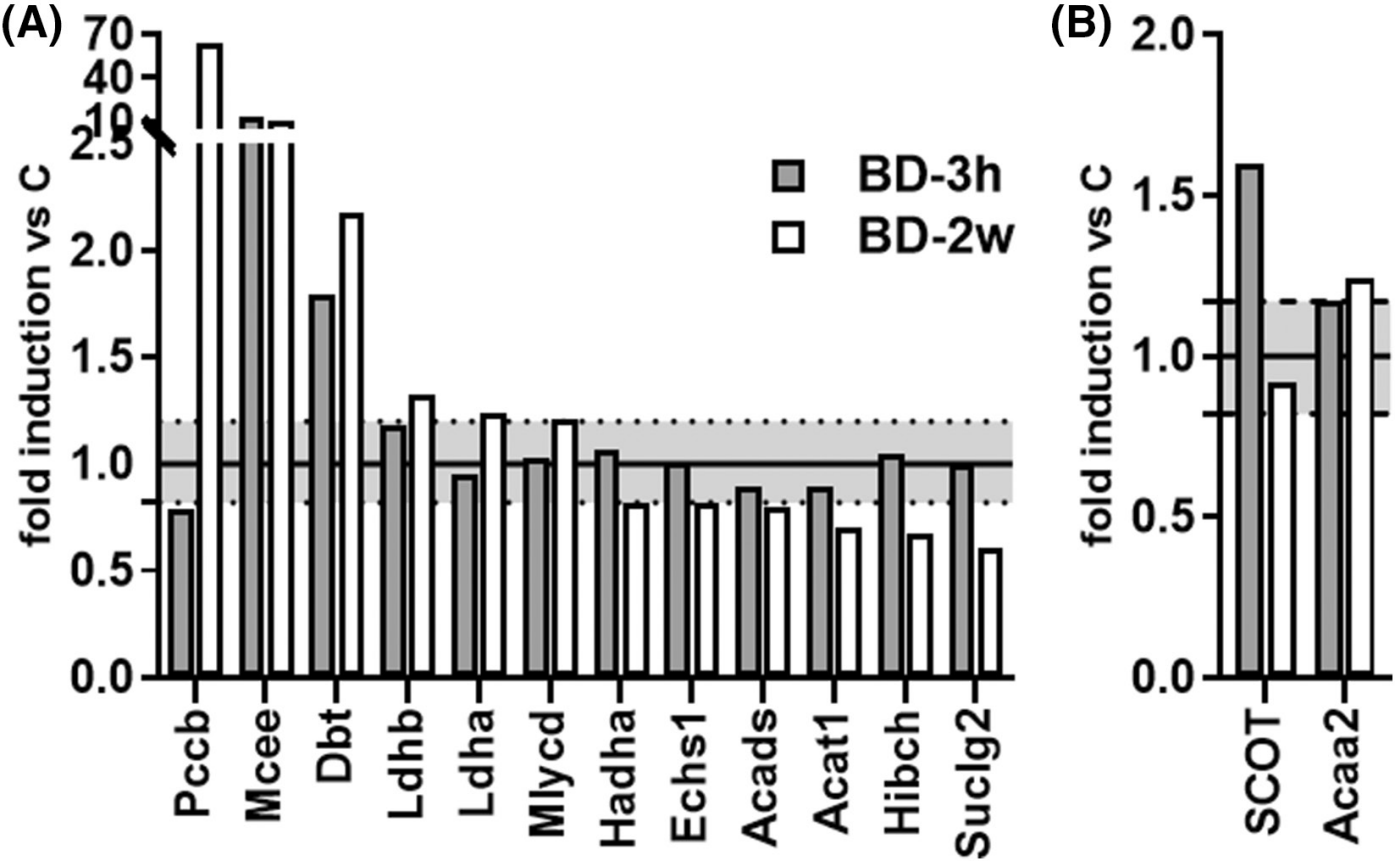
Effect of BD on the levels of proteins involved in propanoate metabolism (A) and ketone bodies metabolism (B). Relative abundance of iBAT mitochondrial proteins involved in propanoate metabolism affected by BD treatments. Gene names of changed proteins have been reported. The values with a fold >1.2 are an expression of upregulation versus C, whereas those with a fold <0.82 are indicative of a downregulation versus C. The upper dotted line represents the value 1.2, and the lower dotted line represents the value 0.82.

According to KEGG analyses some proteins regulated by BD belong to the network of propanoate metabolism (see File S1 and Figure S3). BD treatment for 2 weeks increased the levels of enzymes involved in Propionyl‐CoA metabolism, leading to Succinil‐CoA formation. Specifically, Propionyl‐CoA carboxylase beta chain (Pccb) and Methylmalonyl CoA epimerase (Mcee) exhibited significant fold changes in BD‐2w compared to C, with a remarkable 64‐fold and 13‐fold increase, respectively (Figure 5A). The effect of BD on Methylmalonyl CoA epimerase protein expression was already observed within 3 h from its administration (Figure 5A). On the other hand, levels of enzymes involved in the pathways leading to Acetyl‐CoA from propionyl CoA are reduced by 2 weeks BD treatment, among these are: Short‐chain specific acyl‐CoA dehydrogenase (Acads), Enoyl‐CoA hydratase (Ecsh1), Trifunctional enzyme subunit alpha (Hadha), 3‐hydroxyisobutyryl‐CoA hydrolase mitochondrial (Hibch) (Figure 5A).

BD treatment enhances levels of enzymes involved in ketone bodies metabolization such as Succinyl‐CoA:3‐Ketoacid CoA transferase (SCOT) within 3 h and 3‐ketoacyl‐CoA thiolase (Acaa2), that resulted slightly enhanced in BD‐2w compared to C (Figure 5B).

### 3.5 BD rapidly enhances mitochondrial respiration rate by affecting the activity of the respiratory chain and UCP1

**FIGURE 6:**
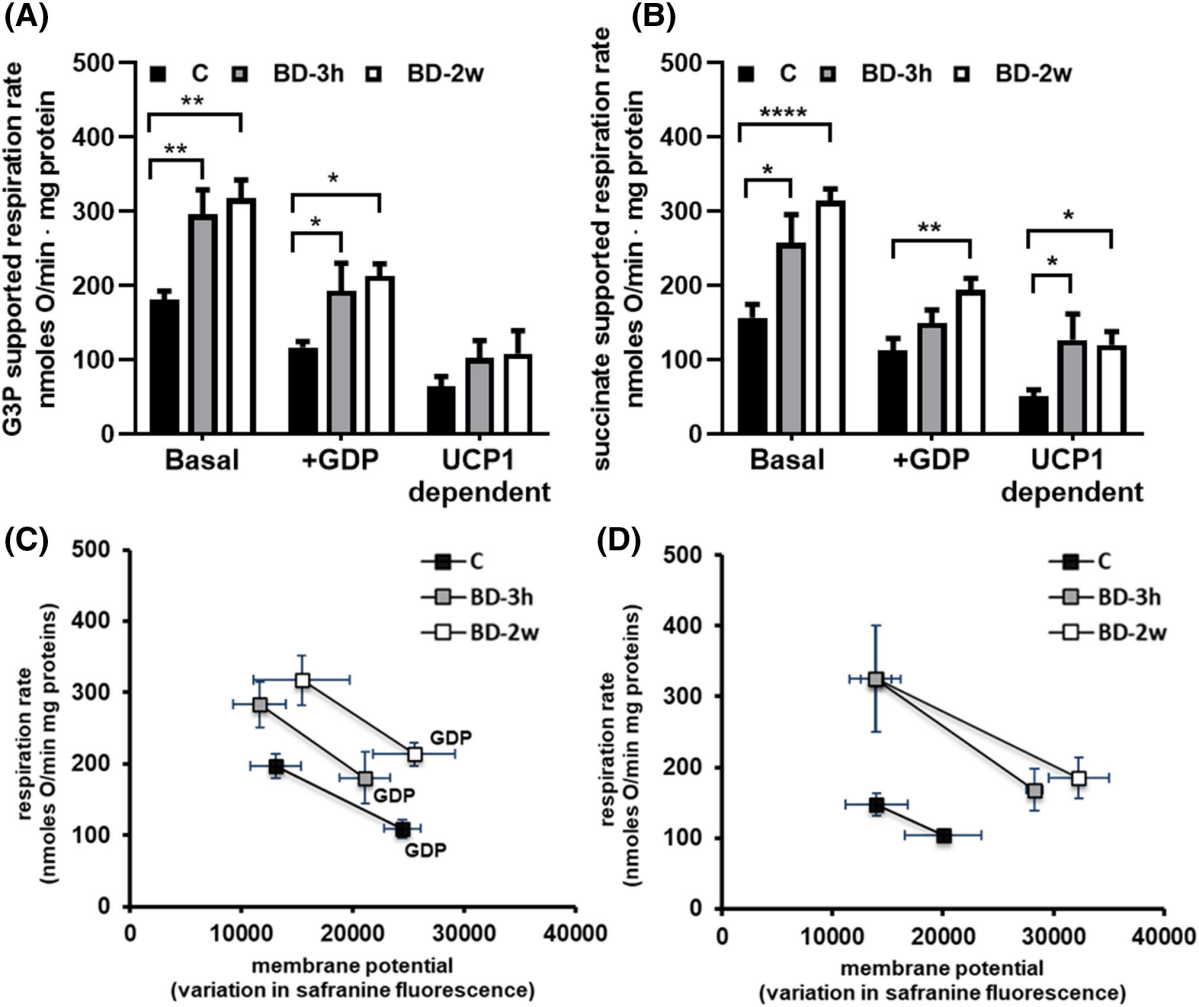
Effect of BD on mitochondrial respiration and the overall activity of reactions involved in the oxidation of substrates. To detect mitochondrial respiration, mitochondria were energized with either G3P (A) or succinate (B). Respiration was evaluated in the absence or the presence of GDP (2 mM). UCP1‐dependent respiration was obtained by subtracting GDP‐inhibited respiration from basal respiration. Values represent the mean ± SEM of 7–8 different rats. Measurements were performed in duplicate. *p < .05, **p < .01, ****p < .0001. To detect the overall activity of reactions involved in the oxidation substrates, membrane potential and mitochondrial respiration were detected in parallel experiments in mitochondria energized with G3P (C) or succinate (D), in the absence (basal) and the presence of GDP. Values represent the mean ± SEM of 5 different samples.

To assess the impact of BD on iBAT mitochondrial functionality, we measured oxygen consumption by using two different respiratory substrates: glycerol‐3‐phosphate (G3P) (Figure 6A) and succinate (Figure 6B). BD treatment leads to an increase in mitochondrial respiration regardless of the respiratory substrate used. This effect was observed within 3 h from BD administration and persisted after 2 weeks of treatment. Specifically, compared to the C group, mitochondria from the BD‐3h group exhibited higher respiration rates, with a similar increase (+64%) in the presence of G3P (Figure 6A) or succinate (Figure 6B) as substrates. The administration of BD for 2 weeks led to increases in respiration rate of +74% and +100% versus C, in the presence of G3P or succinate, respectively.

As expected, the addition of GDP to inhibit the UCP1 activity resulted in a reduction in mitochondrial respiration rate. However, mitochondria from the BD‐treated rats still showed a higher respiration rate compared to the C group, even in the presence of GDP. When looking at UCP1‐linked respiration (obtained by subtracting GDP‐inhibited respiration from basal respiration), BD treatment enhanced it, and the stimulatory effect reached statistical significance in the presence of succinate as a respiratory substrate.

The ability of BD to stimulate respiration, even when UCP1 activity is inhibited by the presence of GDP, suggests that BD may affect the reactions involved in substrate oxidation. To explore this possibility, we evaluated the impact of BD treatment on the overall kinetic of the reactions involved in the oxidation of substrates and in the genesis of proton motive force, including the respiratory chain (Figure 6C,D). When comparing the plots obtained from the three groups, we observed distinct patterns between the BD‐3h and BD‐2w groups compared to C: the plots for BD‐3h and BD‐2w do not overlap with those for C and indicate a change in the overall substrate oxidation kinetics in the former two groups relative to C. In other words, at any given mitochondrial‐membrane potential, mitochondria from the BD‐3h and BD‐2w groups showed a higher respiration rate. This indicates an increase in respiratory chain activity induced by BD administration. Noteworthy, this effect was observed after 3 h and persisted after 2 weeks of treatment. Data from Figure 6D also indicate that a process of proton leak is stimulated by BD treatment, in fact in basal condition, when UCP1‐mediated proton leak is active, mitochondria from BD‐2w and BD‐3h groups respirate more to maintain the same membrane potential.

### 3.6 Ketone bodies induce Kbhb of mitochondrial proteins

**FIGURE 7:**
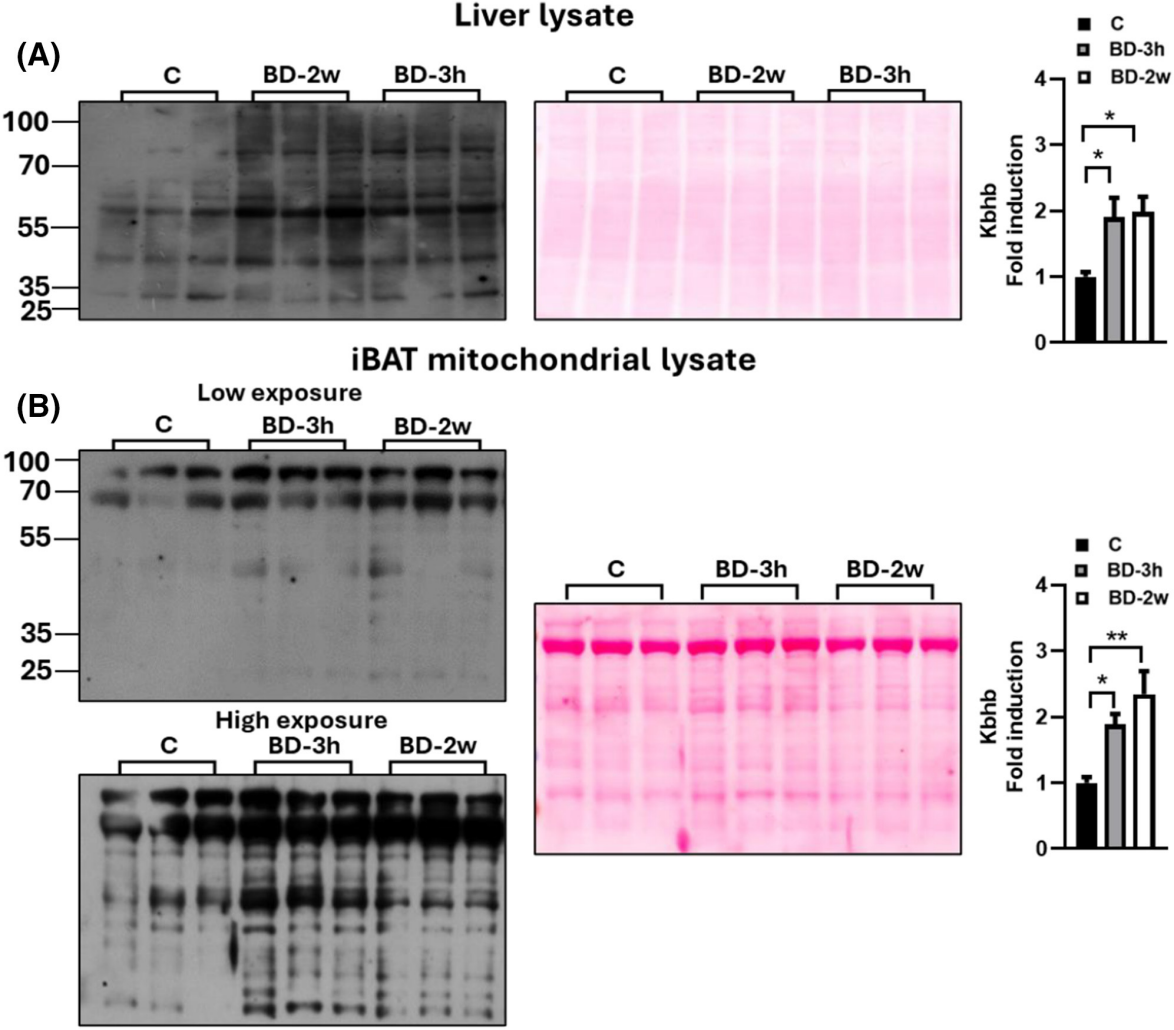
Effect of BD on lysine residues beta‐hydroxybutirylation. Western blot analysis of total lysine beta‐hydroxybutyrylation in the liver lysate (A) and isolated mitochondria (B) using a pan‐K‐bhb antibody. For iBAT mitochondria lysates, two different exposure times were represented. Histograms represent the quantification of data. Ponceau staining was used as a loading (25 μg of protein/rat/lane). Data were normalized to the value obtained for control animals, set as 1. Values represent the mean ± SEM of 5–6 different rats. *p < .05, **p < .01.

We then investigated the Kbhb on mitochondrial proteins by combining Western blot and MS data. Western blot analysis was performed by using an antibody against β‐hydroxybutyryl‐lysine residues, and we used the liver homogenate as a positive control. In liver lysate, we revealed an increase in Kbhb proteins in BD‐3h and BD‐2w versus C (+127% and +134%, respectively), thus confirming data already reported in the literature,^19^ and extending the effect observed to a short‐term (within 3 h). Interestingly, we also observed an increase in protein Kbhb in iBAT mitochondria from BD‐3h and BD‐2w groups versus C (Figure 7). These results led us to evaluate if some subunits of respiratory chain complexes and UCP1 could undergo Kbhb, by proteomics approach based on mass spectrometry (see methods). Indeed, as reported in Table 2, some specific subunits of respiratory chain complexes I (Nufa9, Nufa10, Nufs4), II (Sdhb), III (Uqcrfs1, Uqcrc2), and V (Atp5f1c) displayed lysine residues modified with Kbhb in the BD‐3h group, while UCP1 presents two lysine residues that undergo to Kbhb.

**TABLE 2:** List of the proteins presenting β‐hydroxybutyryl lysine, detected in iBAT mitochondria extracts from C, BD‐3h and BD‐2w samples.

Gene name	Protein description	Peptide sequence	Peptide modifications	C	BD3h	BD 2w
Ndufa9	NADH dehydrogenase [ubiquinone] 1 alpha subcomplex subunit 9, mitochondrial	SKAVGEKEVR	2 beta‐hydroxybutyryl (K)		●	
Ndufa10	NADH dehydrogenase [ubiquinone] 1 alpha subcomplex subunit 10, mitochondrial	KGDPHEMKVTSAYLQDIEDAYKK	beta‐hydroxybutyryl (K)			●
Ndufs4	NADH dehydrogenase [ubiquinone] iron–sulfur protein 4, mitochondrial	NNMQSGVNNTKKWK	2 beta‐hydroxybutyryl (K)		●	
Sdhb	Succinate dehydrogenase [ubiquinone] iron–sulfur subunit, mitochondrial	MAAVVGVSLK	beta‐hydroxybutyryl (K)		●	
Uqcrfs1	Cytochrome b‐c1 complex subunit Rieske, mitochondrial	SSKESSEAR	beta‐hydroxybutyryl (K)		●	
Uqcrc2	Cytochrome b‐c1 complex subunit 2, mitochondrial	GGLGLAGAKAK	beta‐hydroxybutyryl (K)	●	●	
Atp5f1c	ATP synthase subunit gamma, mitochondrial	AEIKGPEDKK	beta‐hydroxybutyryl (K)			●
Atp5f1c	ATP synthase subunit gamma, mitochondrial	GLCGAIHSSVAKQMKNDMAALTAAGK	beta‐hydroxybutyryl (K)			●
Ucp1	Mitochondrial brown fat uncoupling protein 1	GVLGTITTLAK	beta‐hydroxybutyryl (K)		●	●
Ucp1	Mitochondrial brown fat uncoupling protein 1	KELMKSR	beta‐hydroxybutyryl (K)		●	

*Note*: The symbol • indicates the presence of the modified peptide in the samples.

## 4 DISCUSSION

The manipulation of BAT's energy‐burning capacity has the potential to yield metabolic benefits, making it an attractive target for interventions against dysmetabolic diseases and obesity,^2^, ^3^, ^5^ although the current contribution of BAT to whole‐body energy expenditure in humans is still a matter of dispute.^7^, ^8^

The findings presented in this study demonstrate that the administration of BD and the resultant “exogen ketosis” significantly impact iBAT functionality, with mitochondria being a target. In fact, the exposure of the tissue to elevated levels of β‐OHB for 2 weeks leads to an increase in tissue weight/body weight ratio and a change in tissue morphology, characterized by the presence of smaller adipocytes, that are indicative of tissue thermogenesis activation. These findings are in line with results in the literature, reporting that other treatments able to induce ketosis (ketogenic diet or administration of ketone esters) were able to affect the functionality of such tissue.^27^, ^29^, ^40^ However, in studies using ketone ester to induce exogenous ketosis, the treatment resulted in β‐OHB levels about six‐fold the control values.^29^, ^40^ In our study, BD treatment for 2 weeks was able to induce “mild” ketosis, given the evidence that β‐OHB plasma levels in this group were about 0.5 mM, i.e. a value half of those observed following 16 h of food removal (see Figure S4). This suggests that variations in β‐OHB levels within the physiological range are effective in activating iBAT, although circulating β‐OHB concentrations alone provide limited information without the understanding of the tissue's clearance.

The ketogenic diet and ketone ester treatments, above cited, have been reported to enhance the levels of proteins related to respiratory complexes and UCP1 in total BAT lysate, as well as proteins involved in mitochondrial biogenesis,^27^, ^28^, ^40^ thus suggesting an increase in mitochondrial mass. Starting from this knowledge, we decided to give further insight into the effect of β‐OHB on mitochondria by evaluating its influence on the biochemical and bioenergetic parameters of these organelles. Thus, we examined alterations in mitochondrial proteomic patterns associated with changes in mitochondrial functionality. In addition, for the first time, we reported that some of the effects induced by BD on iBAT manifest within 3 h from its in vivo administration. This suggests the direct role of β‐OHB on the tissue, independent of the pleiotropic effects associated with physiological or pathological conditions that increase its levels, such as fasting, ketogenic diet, physical exercise, or diabetes.

Functional data along with Western blot and the proteomic approach revealed that the primary adaptation associated with BD‐induced exogenous ketosis is the activation of mitochondrial respiratory chain functionality and UCP1‐mediated proton leak. Concerning the respiratory chain, BD administration affected the levels of subunits belonging to each of the five respiratory complexes as revealed by western blot and confirmed by proteomic analysis. The last suggested that the regulation of specific subunits within each complex was not uniform, indicating changes in complex composition and suggesting critical roles for certain subunits. Notably, the response of individual subunits to BD administration was highly dynamic, with modulation of some subunits observed within 3 h from its administration. Although the upregulation of specific subunits persisted or increased after 2 weeks of BD treatment, the downregulation of subunits belonging to complexes I and IV was only evident in the short term (BD‐3h group) and disappeared after 2 weeks of treatment. This highlights the markedly dynamic nature of the response of the BAT mitochondrial respiratory chain to elevated levels of β‐OHB. The dynamic up‐and‐down‐regulation of specific respiratory complex subunits appears to be a common process involved in triggering the rapid remodeling of the mitochondrial respiratory chain. In fact, this phenomenon also occurs following in vivo administration of succinate, known to rapidly influence the functionality and proteomics of BAT, in a time interval between 30 min and 3 h.^41^

Another important data emerging from our research is that treatment with BD increased the expression of proteins associated with the structural organization of the mitochondrial respiratory chain, among these, succinate dehydrogenase assembly factor (Sdhaf1), which facilitates the assembly of complex II,^42^ and Cox7a2l protein, involved in the stabilization of complexes III and IV, by promoting their supramolecular organization in III_2_ + IV supercomplex.^43^

BD administration induces post‐translational Kbhb of mitochondrial proteins, as revealed by Western blot analysis (Figure 7). In line with this, proteomic data allowed us to identify Kbhb modifications in three subunits of complex I (Ndufa9, Ndufa10, Ndufs4, important for the assembly and the stability of the complex), one subunit of complex II (Sdhb, which is part of the catalytic core of the complex), one subunit of complex III (Uqcrfs1, part of the catalytic core of the complex), one subunit of complex V (Atp5f1c or gamma subunit, involved in rotatory catalysis),^42^ as well as in UCP1. Similar to other mitochondrial post‐translational modifications involving acylation process, Kbhb modifications could influence protein function and protein–protein interactions.^44^ Therefore, the Kbhb modifications discussed here may be part of the mechanism involved in the dynamic responsive behavior of the respiratory chain and UCP1 to β‐OHB.

The mitochondrial functional analysis reported here is in line with and corroborated by Western blot and proteomic analysis. In fact, by evaluating mitochondrial respiration rate, we were able to demonstrate the ability of BD to rapidly increase it, with the activation of the respiratory chain and UCP1 being involved. Indeed, when iBAT mitochondrial respiration is evaluated in the basal condition, its control is shared between proton leak (in BAT mainly mediated by UCP1) and the overall reactions involved in the oxidation of the substrate and in the generation of proton motive force (among these the respiratory chain). However, in the presence of GDP, which abolishes the contribution of UCP1‐mediated proton leak, the control exerted by the respiratory chain on respiration becomes more relevant.^45^ The ability of BD to enhance mitochondrial respiration rate both under basal conditions and in the presence of GDP, reported here, indicates an improvement in respiratory chain activity. This is further supported by the experiment aimed at determining the overall kinetics of the reactions involved in substrate oxidation and proton motive force generation, which was enhanced by BD treatment. This activation also contributes to mitochondrial thermogenesis, since BAT UCP1 dissipates the proton motive force, generated by the respiratory chain, as heat.

Furthermore, despite observing a BD‐dependent activation of the respiratory chain, in mitochondria respiring under basal conditions we did not find a higher mitochondrial inner membrane potential. This indicates that the proton motive force is rapidly dissipated through the proton leak process (see Figure 5B).

The increase in UCP1 proteins, observed within 3 h from BD administration and even more prominent after 2 weeks of treatment, together with BD‐induced enhancement in UCP1‐dependent respiration, supports the involvement of such protein in the activation of mitochondrial respiration and thermogenesis.

Another aspect emerging from our data is that BD administration promoted an increase in mitochondrial levels of proteins involved in succinyl‐CoA formation from propionyl‐CoA, whereas reducing the levels of proteins involved in the formation of Acetyl‐CoA from Propionyl‐CoA. This suggests that BD treatment enhances the formation of succinyl‐CoA and thus succinate, whose utilization as mitochondrial respiratory substrate was increased by the same treatment, as revealed by increased succinate‐supported respiration (Figure 6B). Detailed metabolic fluxes analysis will be necessary to validate this possibility.

A further important point to be considered is that the formation of succinyl‐CoA plays also an important role in ketolysis in extra‐hepatic tissues, since it provides CoA for the formation of acetoacetyl‐CoA from the ketone body acetoacetate. This reversible reaction is catalyzed by succinyl‐CoA:3‐ketoacid CoA transferase^10^, ^46^; subsequently, a mitochondrial 3‐ketoacyl‐CoA thiolase converts acetoacetyl‐CoA to two molecules of acetyl‐CoA, which can enter the tricarboxylic acid cycle. Therefore, the ability of BD to increase the levels of proteins involved in the pathways leading to succinyl‐CoA may also be useful for β‐OHB metabolization. This possibility agrees with our results showing that BD treatment increased succinyl‐CoA:3‐ketoacid CoA transferase (observed within 3 h) and slightly 3‐ketoacyl‐CoA thiolase levels.

It should be mentioned that our study has some limitations. First, all the results reported here referred to male rats, and we did not investigate for potential sexual dimorphism response of BAT to BD administration.

Concerning proteomic analysis, pooling the samples does not allow us to consider the variability between biological samples and that for the proteins, whose levels were not validated by western blot, there is a possibility that an outlier sample drives their variation.

In the present paper, we did not investigate whether the variation in BAT activity could have affected the whole animal energy balance and to which extent the reduction in food intake, due to BD treatment, could have contributed to the decrease in body weight gain and even more in gonadal WAT mass. We limited our observation to the whole animal oxygen consumption of a subset of animals, which was stimulated (Figure S1). Further detailed studies are needed to address this fundamental aspect, given the evidence that in the literature discordant opinions are presented on the ability of exogen‐induced ketosis to affect whole animal energy expenditure.^29^, ^40^, ^47^

**FIGURE 8:**
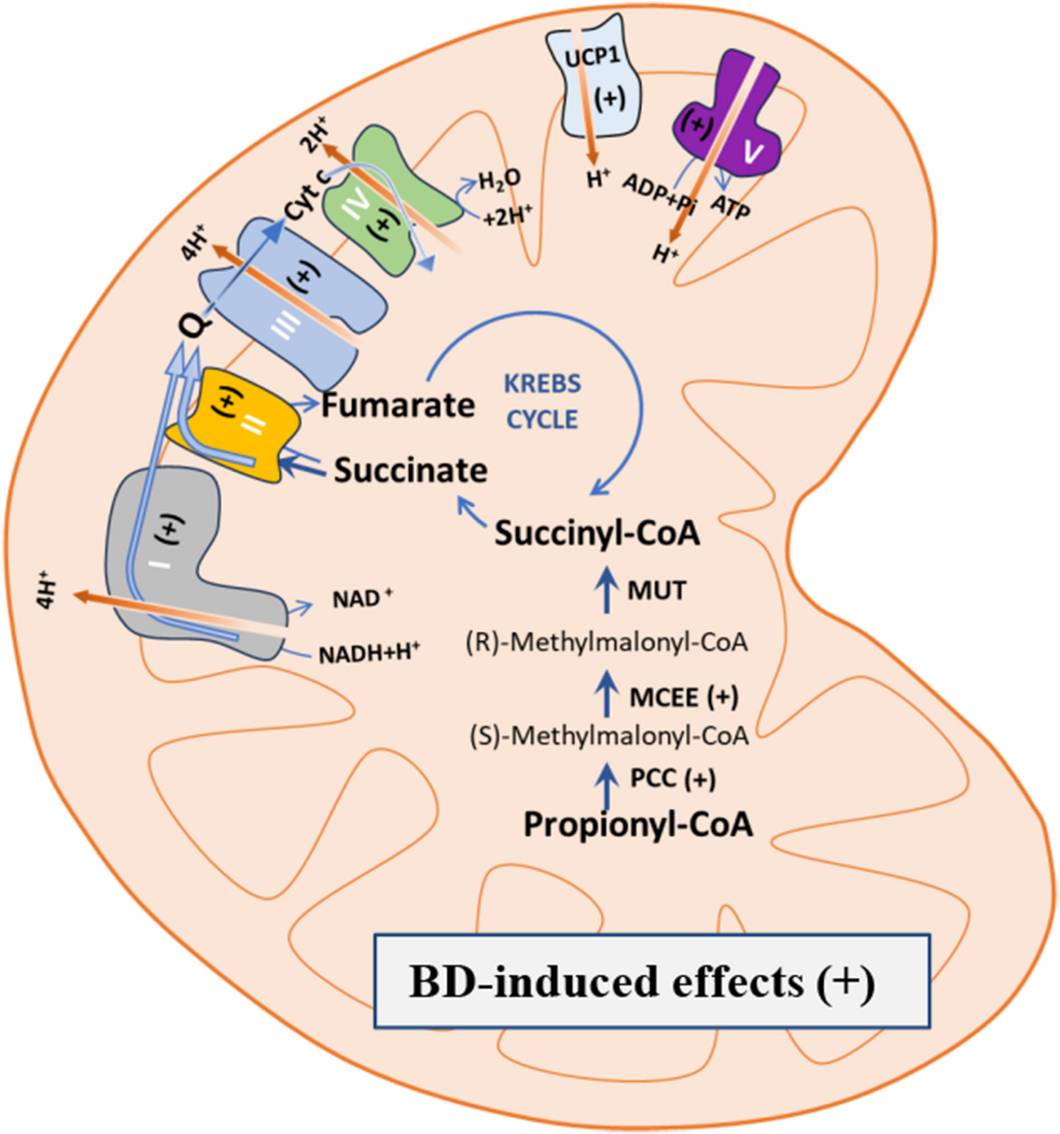
Schematic representation of the events underlying the effect of BD on iBAT mitochondrial functionality. BD treatment stimulates BAT mitochondrial respiration by improving the activity of the respiratory chain and UCP1. Increases in specific subunits of the five respiratory complexes and UCP1 underlie the BD effect on mitochondrial respiration. BD also increases the levels of proteins involved in the anaplerotic pathway that forms succinyl‐CoA from propionyl‐CoA. Succinyl‐CoA is the precursor of succinate, the substrate of complex II of the respiratory chain, and BD enhances the use of succinate as a respiratory substrate. The symbol +, when under parentheses, indicates that one or more subunits of the specific respiratory complexes or other indicated proteins were upregulated by the BD treatment.

In conclusion, by taking advantage of the ability of BD to increase plasma levels of β‐OHB, we suggest a role for β‐OHB as a signaling molecule that can rapidly affect iBAT physiology/thermogenesis. The findings reported in the present paper provide valuable insights into the molecular mechanisms by which β‐OHB influences iBAT mitochondrial function and shed light on its effects. In particular, the mitochondrial respiratory chain, UCP1, and pathways involved in propionyl‐CoA metabolism are targets of β‐OHB. A schema/picture of the events underlying the mechanism by which BD affects iBAT mitochondrial functionality is reported in Figure 8. The ability of β‐OHB to rapidly induce Kbhb of mitochondrial proteins suggests a direct effect of the molecule on the organelle.

This finding opens new opportunities for further studies aiming to assess the actual impact of Kbhb on mitochondrial protein function and BAT mitochondrial bioenergetics, as well as the ability of BD to influence the response of BAT to cold exposure and the whole animal energy balance.

## AUTHOR CONTRIBUTIONS

Assunta Lombardi, Paola Venditti, Angela Amoresano, and Pieter de Lange designed research. Paola Venditti, Gabriella Pinto, Giuliana Panico, Rita De Matteis, Vincenzo Migliaccio, Gianluca Fasciolo, Gaetana Napolitano and Stefania Serpico performed researches. Assunta Lombardi, Pieter de Lange founding acquisition, Paola Venditti, Rita De Matteis, Lillà Lionetti, Pieter de Lange, Claudio Agnisola, Angela Amoresano, and Assunta Lombardi analyzed and interpreted data. Assunta Lombardi, Paola Venditti and Gabriella Pinto wrote the original draft. All authors were involved in revising the manuscript.

## DISCLOSURES

The authors declare no conflicts of interest.

## Supporting information


Figures S1–S4.

Dataset S1.

## Data Availability

Data that support the findings of this study are available in the article and the supplementary material.
