# Supplementary material for: Rapid modulation of interscapular brown adipose tissue mitochondrial activity by ketosis induced by 1,3‐butanediol administration to rats
Source: FASEB J. 2024 Nov 22;38(22):e70195. doi: 10.1096/fj.202401592RR (PMC11583952; doi:10.1096/fj.202401592RR)
Supplement: Supplementary file 1 — Figures S1–S4. [file FSB2-38-e70195-s002.pdf]

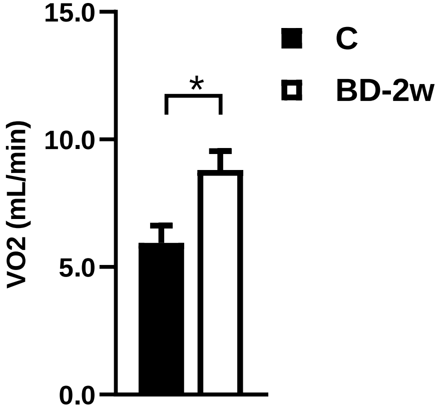

Effect of BD administration for 2 weeks on whole rat metabolic rate
Values represent the mean ± SEM of 3 different rats*p<0.05 vs C

Oxygen consumption (V̇O2) measurements were made using a four-chamber, indirect, open-circuit calorimeter Oxymax system (Pan Lab, Cornella, Barcelona, Spain) with one rat per chamber. After a 1-h period of adaptation to the metabolic chamber, V̇O2 was measured in individual rats at 15-min intervals for 4 h.

Supplementary Fig 1

OXIDATIVE PHOSPHORYLATION

Up-regulated proteins

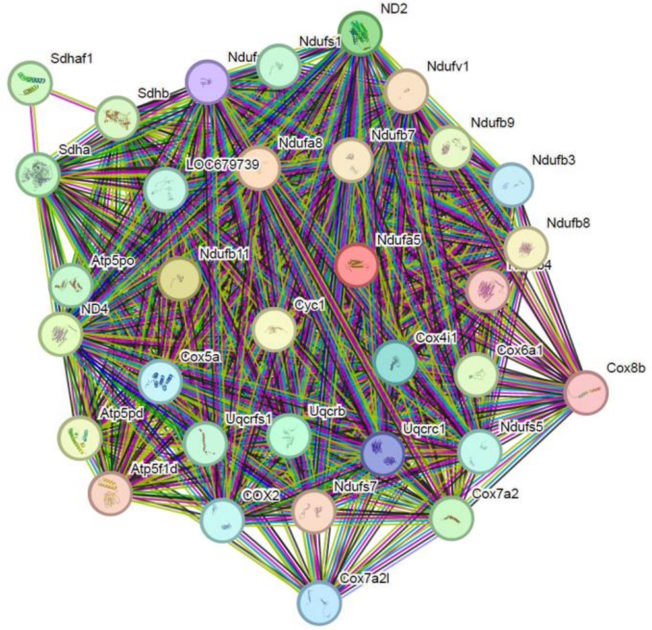

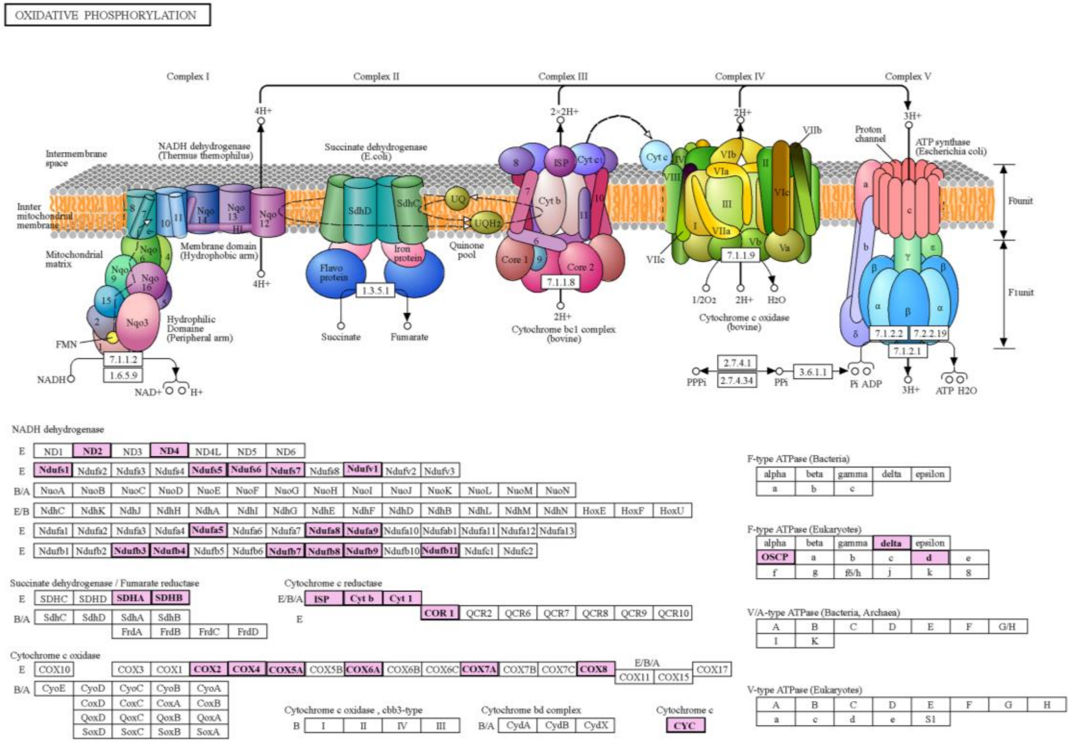

Supplementary Fig.1 String network analysis of regulated proteins involved in oxidative phosphorylation (A) and KEGGs pathways analysis in BD-2w. vs C. The up-regulated proteins are depicted in pink

Supplementary Fig 2

PROPANOATE METABOLISM

Up-regulated proteins

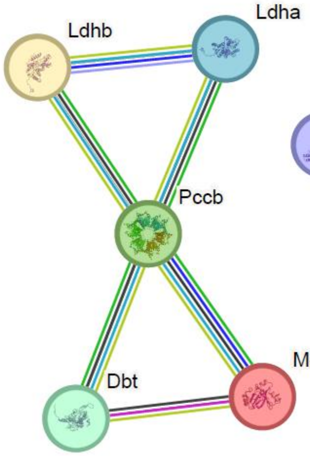

Down-regulated proteins

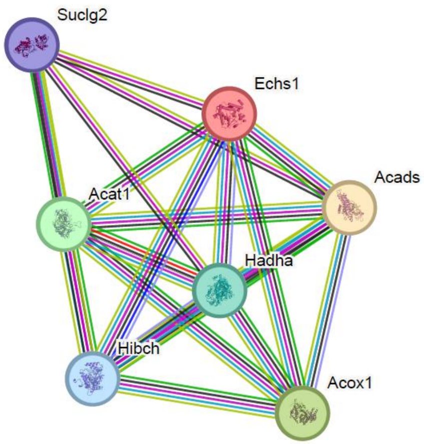

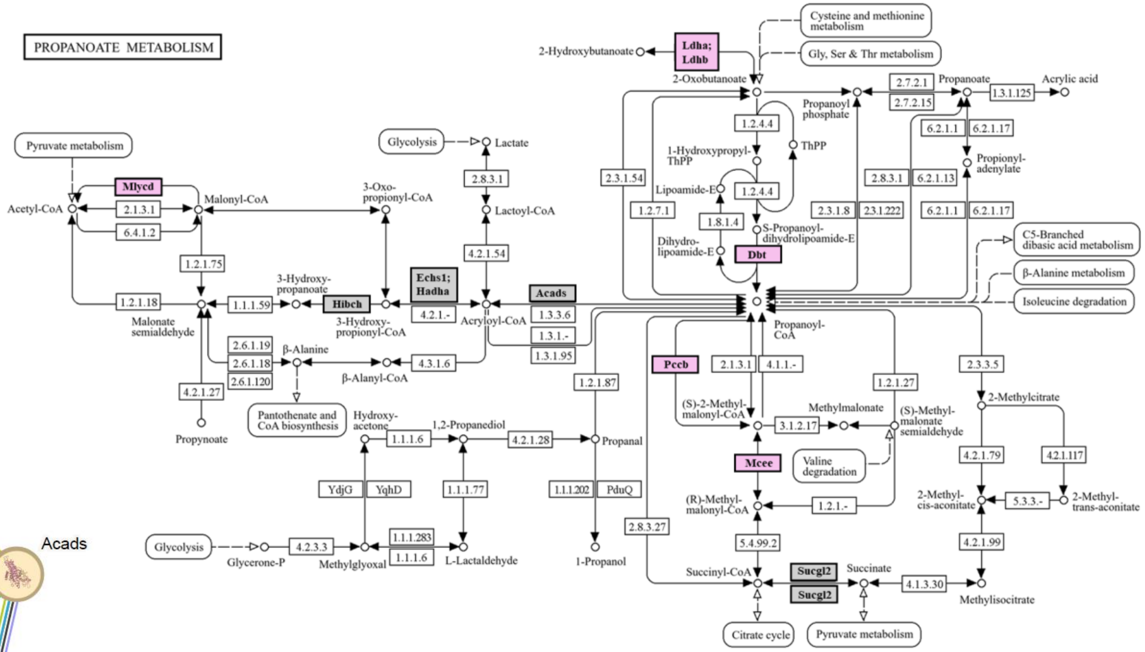

String network analysis (A) and KEGGs analysis (B) of An) of regulated proteins involved in in propaonate metabolismin BD-2w vs C. In the KEGG analysis (B) the up-regulated proteins are depicted in pink, the down-regulated proteins are depicted in grey

Supplementary Fig 3

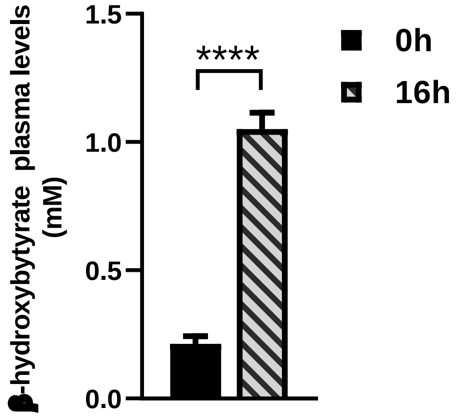

Plasma levels of β-OHB detected in rats after 16 hours of food removal. Values represent mean ± SE of 4 animals. Two-tailed Student test was performed. ****P<0.0001

Supplementary Fig 4
